# Three- and Multi-Phase Extraction as a Tool for the Implementation of Liquid Membrane Separation Methods in Practice

**DOI:** 10.3390/membranes12100926

**Published:** 2022-09-25

**Authors:** Artak E. Kostanyan, Vera V. Belova, Andrey A. Voshkin

**Affiliations:** Kurnakov Institute of General and Inorganic Chemistry, Russian Academy of Sciences, 31 Leninskii pr., 119991 Moscow, Russia

**Keywords:** three- and multi-phase extraction, bulk and supported liquid membranes, purification of wastewaters from phenol

## Abstract

To promote the implementation of liquid membrane separations in industry, we have previously proposed extraction methods called three- and multi-phase extraction. The three-phase multi-stage extraction is carried out in a cascade of bulk liquid membrane separation stages, each comprising two interconnected (extraction and stripping) chambers. The organic liquid membrane phase recycles between the chambers within the same stage. In multi-phase extraction, each separation stage includes a scrubbing chamber, located between the extraction and stripping chambers. The three- and multi-phase multi-stage extraction technique can be realized either in a series of mixer–settler extractors or in special two- or multi-chamber extraction apparatuses, in which the convective circulation of continuous membrane phase between the chambers takes place due to the difference in emulsion density in the chambers. The results of an experimental study of the extraction of phenol from sulfuric acid solutions in the three-phase extractors with convective circulation of continuous membrane phase are presented. Butyl acetate was used as an extractant. The stripping of phenol from the organic phase was carried out with 5–12% NaOH aqueous solutions. The prospects of using three-phase extractors for wastewater treatment from phenol are shown. An increase in the efficiency of three-phase extraction can be achieved by carrying out the process in a cascade of three-phase apparatuses.

## 1. Introduction

Liquid membrane separations (bulk [1,2,3], supported [4,5,6,7,8,9,10,11], emulsion [12,13,14,15,16,17,18,19,20,21,22,23,24,25,26,27,28,29]) combining extraction and stripping processes in a single mass-transfer unit allow the separation of species on their partition between two aqueous phases. Mass transfer occurs through an intermediate organic phase (mass-transfer medium). The organic phase is held stationary in the operating system, while the first (a donor feed) and second (an acceptor stripping) aqueous phases are eluted through it as the mobile phases. These processes provide purification of the aqueous solution and/or concentration of a solute in the second aqueous solution and include the steps of contacting the initial aqueous solution from which it is desired to extract the solute with an organic phase containing, as a rule, a chemical having a high affinity for the solute to be removed. The organic phase enriched in the solute molecules is then brought into contact with the aqueous “stripping” solution, which has a higher affinity for the solute than the chemical in the organic phase. The aqueous feed solution is thus purified of the solute, which is concentrated into the aqueous stripping solution.

In supported liquid membranes, an organic liquid membrane phase is kept in the small pores of a polymer support by capillary forces. The aqueous feed solution and the extract phase move on opposite sides of the porous polymer support impregnated with the liquid membrane. This membrane extraction method is of particular interest because of its stability and simplicity.

Emulsion liquid membranes are complex water-in-oil emulsions formed by organic solvent and surfactant-stabilized water stirred into the aqueous feed solution. Typically, the extract phase is encapsulated as microdroplets in liquid membrane droplets rising (or falling) in the continuous phase of the feed solution. Mass transfer occurs between the aqueous continuous and inner phases through the immiscible membrane phase. This membrane extraction method is practically not very attractive because of the need to prepare and break the emulsion. 

Analysis of mass transfer in three-phase extraction systems (conjugated extraction-stripping, liquid membranes) under various contacting schemes showed [30] that the liquid membrane methods offer advantages over conventional conjugated extraction–stripping when the mass-transfer efficiency of extraction step is substantially higher than that of the stripping. When the mass-transfer efficiencies of both steps are comparable or when the efficiency of stripping is higher, conjugated extraction–stripping demonstrates better results, except for supported liquid membranes. Supported liquid membranes provide higher extraction efficiency; at different mass transfer rates in the extraction and stripping sides, supported liquid membranes with counter current flow of both mobile phases are more efficient than conjugated extraction–stripping, and when they are equal to each other, both processes show the same results.

**Figure 1:**
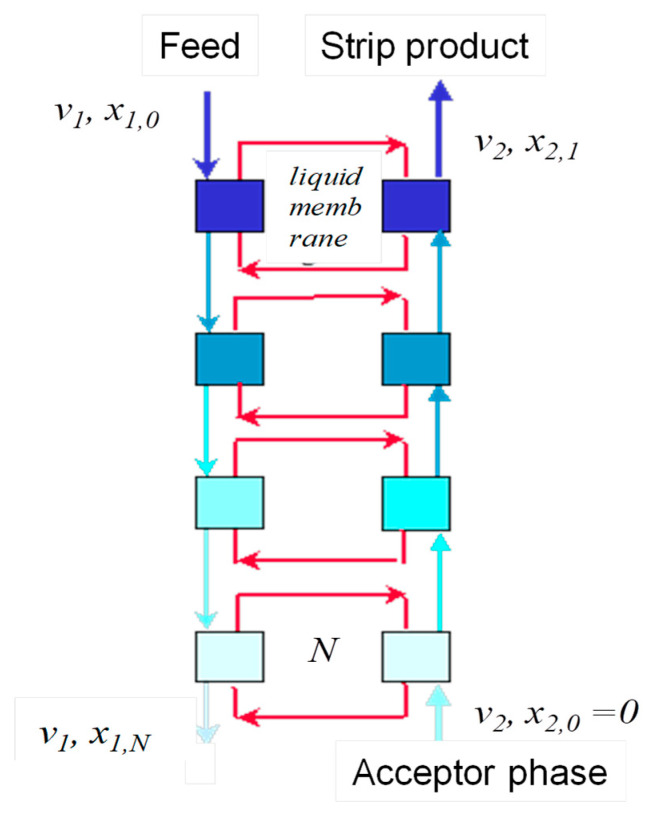
Schematic diagram of three-phase multi-stage extraction processes.

Due to the complexity and low productivity, the above-mentioned conventional liquid membrane methods have not yet found wide industrial application. These shortcomings can be avoided by using three- and/or multi-phase extraction [30,31,32,33,34,35], which can be considered as a modification of bulk liquid membrane technique. Three-phase multi-stage extraction process is carried out in a cascade of N mass-transfer stages (or liquid membrane extraction stages), each comprising two interconnected contact chambers: extraction and stripping chambers (Figure 1). The organic phase (the liquid membrane) recycles between the chambers within the same stage, building N closed circuits. The liquid membrane is thus drawn in a cross-flow with the acceptor and donor phases within the same stage, while the donor and acceptor phases are drawn through all of the stages in the counter current mode. This multi-stage extraction process can be considered as the stagewise embodiment of bulk-supported liquid membrane technique.

The theory for various schemes of the three-phase multi-stage extraction was developed in [30,32,33,34,35,36]. When the equilibrium distribution of the solute that passes from one phase to another is attained in the chambers (each chamber represents a theoretical plate), the process efficiency (the outlet concentration in the raffinate) can be determined by the following equation:

(1) $$\frac{x_{1,N}}{x_{1,0}}=\frac{1-F_{1}F_{2}}{{\left[\frac{1+F_{1}}{\left(1+F_{2}\right)F_{1}}\right]}^{N}-F_{1}F_{2}}$$

where $F_{1}=\nu_{1}/\left(wm_{1}\right)$, $F_{2}=wm_{2}/\nu_{2}$ are the mass-transfer factors in extraction and stripping chambers, respectively; $\nu_{1}$, $\nu_{2}$ are the flow rates of the donor and acceptor phases, and w is the flow rate (circulation rate) of the liquid membrane; $m_{1}=y^{*}/x_{1}^{*}$ and $m_{2}=y^{*}/x_{2}^{*}\;$ are the equilibrium distribution coefficients; $x_{1}$, $x_{2}$, and y are the concentrations of the solute passing from one phase to another in the donor and acceptor phases and in the liquid membrane, respectively, and the symbol * stands for equilibrium conditions. 

As the rate of the liquid membrane circulation between the extraction and stripping chambers rises (w→∞), the solute concentrations in the phases approach equilibrium values and Equation (1) reduces to

(2) $$\frac{x_{1,N}}{x_{1,0}}=\frac{F^{N}-F^{N+1}}{1-F^{N+1}}$$

with $F=\frac{\nu_{1}m_{2}}{\nu_{2}m_{1}}$.

Equation (2) defines the efficiency of the three-phase extraction in a cascade of *N* theoretical three-phase mass-transfer stages.

The three- and multi-phase multi-stage extraction technique can be realized either in a series of mixer–settler extractors [34,35] or in special two- or multi-chamber extraction apparatuses [36,37,38,39,40,41,42].

**Figure 2:**
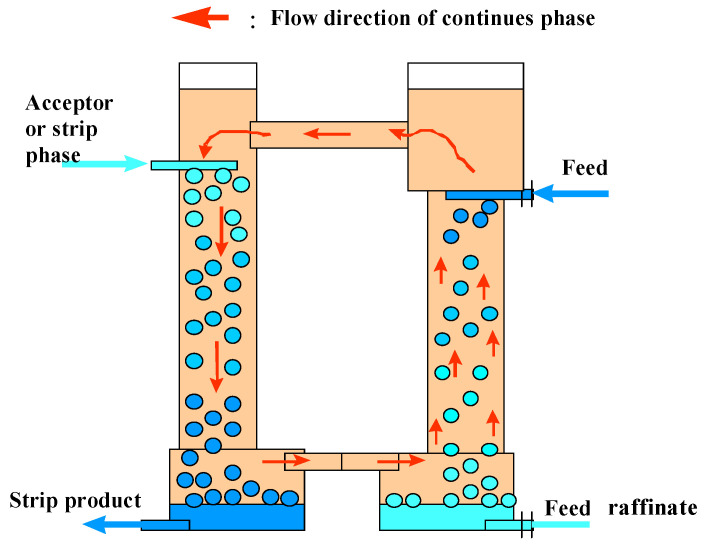
Schematic arrangement of the three-phase extraction apparatus.

In Figure 2 is shown a schematic arrangement of the three-phase extraction apparatus indicating the basic operation and flow patterns of the liquids. The circulation of liquid membrane, which is the continuous phase, between the extraction and stripping chambers (columns) takes place due to the difference in emulsion density in the chambers. When the apparatus is being operated to concentrate a dilute feed solution, the feed flow rate should be greater than the flow rate of the acceptor phase. This will cause circulation between the chambers which is co-current on the feed side and counter-current on the strip side. It is possible to change the circulation direction by operating the strip side at high levels of reflux.

**Figure 3:**
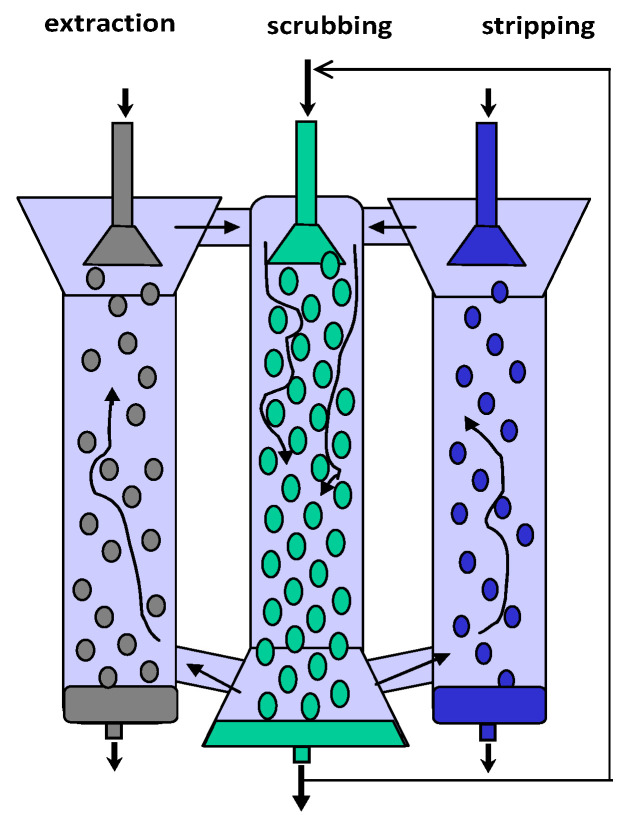
Schematic arrangement of the multi-phase extraction apparatus.

When there are two or more solutes in the feed solution that can be extracted by the liquid membrane, the product in the stripping solution will be contaminated with unwanted solutes extracted from the feed solution. In order to ensure the most efficient counter-current mode in both the extraction and stripping chambers and to minimize the contamination of the stripping stream with the feed stream and vice versa (the mutual contamination of the phases related to the entrainment of small droplets by the circulating continuous phase), in the apparatus shown in Figure 3, a third (scrubbing) chamber is provided, located between the first two ones. In the scrubbing chamber, the stream of loaded continuous membrane phase moving from the feed side and the stream of continuous membrane phase denuded of the extracted solutes by the stripping solution join and are contacted by the scrubbing stream [42]. It is clear that the flow rate of the scrubbing stream must be greater than the flow rates of the feed and stripping streams. In order to minimize the volume of scrubbing solution employed, the scrubbing liquid can be recycled back through the continuous organic phase as shown in Figure 3.

One of the harmful impurities contained in the wastewater of organic industries (for example, synthetic alcohol plants, fragrant substances production) is phenol. Currently, for the purification of wastewaters from phenol and the separation of phenol-containing mixtures, solvent extraction methods are used [43,44].

However, the use of these methods does not always provide the degree of wastewater treatment required by sanitary standards. In this regard, the task of finding a more promising way to treat phenolic wastewater is topical. This work is devoted to an experimental study of the process of wastewater treatment from phenol in three-phase extractors.

## 2. Materials and Methods

### 2.1. Apparatuses

**Figure 4:**
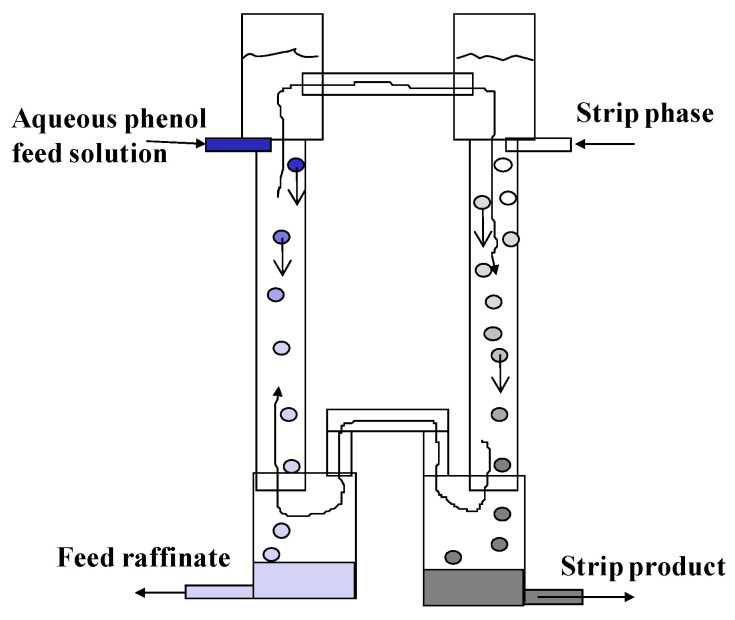
Schematic arrangement of the experimental small three-phase extraction apparatus.

Two three-phase extractors of different sizes were designed and made of glass. A small apparatus consisting of two columns (extraction and stripping) with a diameter of 30 mm and a height of 400 mm, connected at the top and bottom by overflow pipes, is shown in Figure 4.

**Figure 5:**
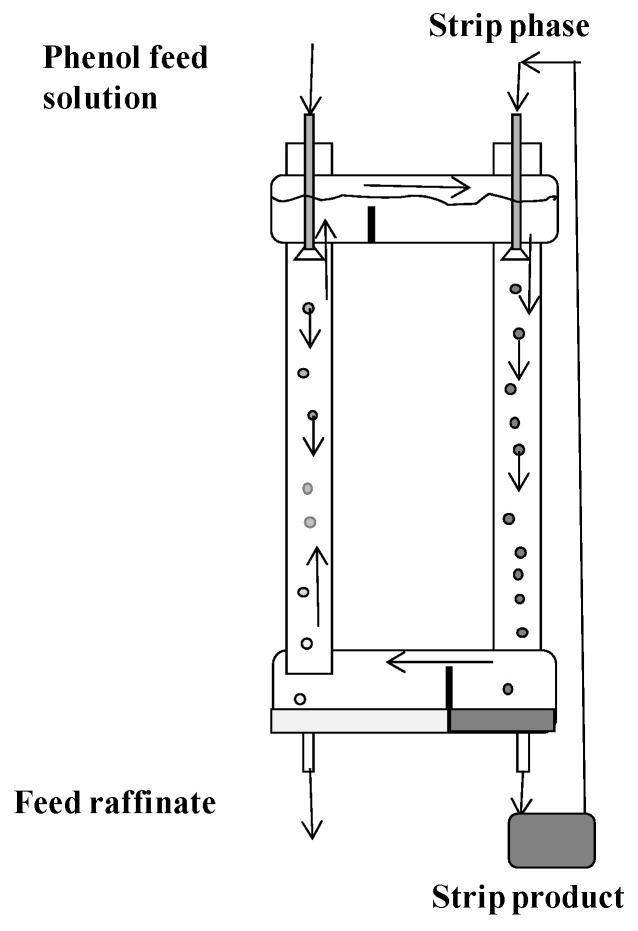
Schematic arrangement of the experimental large three-phase extraction apparatus.

The columns were equipped with bottom settlers (diameter 50 mm) and upper separating zones (diameter 40 mm). A schematic diagram of a large three-phase extractor is shown in Figure 5.

In this apparatus, the extraction and stripping chambers are connected at the bottom by a common horizontal cylindrical settling tank (diameter 80 mm), and at the top by a separator of a similar design (column height 900 mm, diameter 30 mm). The volume of the organic phase in the small apparatus was 0.85–0.9 L, and in the large apparatus it was 3.4 L. Dispersion of aqueous phases in the chambers in both apparatuses was carried out using fluoroplastic distributors with hole diameters (*d_e_* and *d_s_*) of 1, 2, and 3 mm.

### 2.2. Feed Solution and Extraction System

Model aqueous and sulfuric acid phenol-containing solutions were used for research. Butyl acetate was used as an extractant (organic phase) for extracting phenol from aqueous solutions. The concentration of phenol in the aqueous phase was determined by the spectroscopic method. Table 1 shows equilibrium data for the extraction system water–phenol–butyl acetate. The concentration of phenol in the organic phase was determined from the difference between its concentrations in the initial solution and in the aqueous phase after extraction.

As follows from the data given in Table 1, the distribution coefficient of phenol does not depend on its concentration and the acidity of the aqueous phase. A 5–12% NaOH solution was used as a stripping phase. 

### 2.3. Experimental Procedures

The operating principle of three-phase phenol extraction is as follows: the three-phase apparatus is filled with the organic phase (butyl acetate), the initial aqueous phenol solution is fed into the extraction chamber, and an aqueous solution (the stripping phase) is fed into the stripping chamber. After crushing with dispersants, the aqueous phases pass through the continuous organic phase in the form of a stream of droplets, coalesce in settling tanks with the formation of layers of the corresponding phases, and then are removed from the apparatus through U-shaped devices. Due to the difference in emulsion densities in the chambers, the continuous organic phase circulates between the extraction and stripping chambers. As a result, there is a co-current flow of the aqueous and organic phases in one chamber and a counter-current flow in the other chamber. The density of emulsions in the chambers is determined by the retention (holdup) of the respective dispersed phase (the fraction of the volume of the column occupied by this phase) and its density. Retention of dispersed phases can be controlled by changing the droplet size and phase flow rate.

The stripping phase (NaOH solution) was circulated at constant rate *v_s_* through the intermediate vessel and the stripping chamber as shown in Figure 5. Thus, during the experiment, the bound phenol accumulated in the stripping phase. The aqueous feed solution with the concentration of phenol *x_f_* was fed into the extraction chamber at a constant flow rate *v_f_*. In the extraction chamber, the countercurrent movement of the phases was maintained, and in the stripping chamber, the co-current movement of the phases was maintained. After reaching the stationary mode of operation (when a constant concentration of phenol in the raffinate was established), the concentration in the raffinate (*x_r_*) leaving the extraction chamber was measured. Although the concentration in the stripping phase can be determined from the material balance equations, in some experiments, the concentration of bound phenol in the stripping phase (*x_s_*) was also measured at the end of the experiment.

## 3. Results

The results of experiments carried out on small and large apparatuses are given in Table 2 and Table 3. In experiments no. 4–7 (Table 2), four sieve plates were placed in the extraction chamber. In these experiments, droplet coalescence was observed over the plates. In experiments no. 1, 2, and 4, the 5% NaOH solution was used as the stripping phase, and in the rest, 12% NaOH was used.

In experiments no. 11–14 (Table 3), intense coalescence of droplets of the feed aqueous phase was observed in the upper part of the extraction chamber. In experiment no. 12, large drops occupied the entire section of the chamber, so the degree of phenol extraction was low. In experiments no. 15 and 16, nine vibrating sieve plates were placed in the extraction chamber. The oscillatory movement of the plates prevented the coalescence of droplets, resulting in a significant decrease in the phenol content in the raffinate.

The efficiency of the three-phase extraction in the the experimental apparatuses, estimated by the value of the degree of extraction *E* = 1 − *x_r_*/*x_f_*, depends on the efficiency of mass transfer in the chambers and the rate of circulation of organic phase between them. The efficiency of mass transfer in the chambers is determined by holdup of the respective dispersed phase, the droplet size, phase flow mode (co-current or counter-current), and rate. When the transferred component undergoes a rapid and irreversible conversion in the stripping phase, as occurs in the isolation of phenol from aqueous solutions, the efficiency of the co-current and counter-current flow designs is the same, and the rate of the three-phase extraction process is determined by the rate of mass transfer in the extraction chamber (in this case, in Equation (2) *m*_2_ = 0). This circumstance makes it possible to concentrate the phenolate in the stripping phase by recycling this phase at the stripping stage. At the same time, the degree of extraction of phenol in the extraction chamber remains constant and depends only on the efficiency of the extraction. 

As the rate of mass transfer in the extraction chamber and the rate of circulation of the organic phase between the chambers increase, the concentration of phenol in the raffinate according to Equation (2) should approach zero. This is confirmed by experiments 1, 15, and 16 in the large apparatus, in which, thanks to the new design of the overflows, a high circulation rate of the organic phase was provided.

On the basis of the conducted experimental studies, the following conclusions can be drawn:

It seems promising to use three-phase extractors for wastewater treatment from phenol. Using the recycle of the stripping phase with an excess alkali content, it is possible to achieve multiple (10-fold or more) concentrations of phenolate in the stripping phase.

The rate of the phenol extraction process is determined by the rate of mass transfer in the extraction chamber. To intensify the mass transfer at the extraction stage, more efficient dispersing devices, such as stirrers, can be used.

A further increase in the efficiency of three-phase extraction can be achieved by carrying out the process in a cascade of three-phase apparatuses.

The main advantage of three-phase extraction over conventional liquid membrane techniques is that three-phase extraction is based on conventional solvent extraction equipment (mixer–settler extractors and extraction columns), which facilitates the application of the liquid membrane principle in industrial extraction separation technologies.

## Figures and Tables

**Table 1:** Results of phenol extraction with butyl acetate.

No	pH	Phenol Concentration, ppm		Distribution Coefficient
		Aqueous Phase	Organic Phase	
1	7	780	43,630	55.9
2	7	460	20,600	44.8
3	7	81	4350	53.7
4	7	58	3070	52.9
5	2.1	40	2020	50.5
6	2.1	20	1030	51.5

**Table 2:** Conditions and results of experiments conducted in the small apparatus.

No	*v_e_*, L/h	*d_e_*, mm	*v*_s_, L/h	*d_s_*, mm	*x_f_*, ppm	*x_r_*, ppm	*E*	*x_s_*, ppm
1	5.3	2	8.5	1	1050	97	0.91	14,900
2	3.3	2	5.3	1	10,600	2280	0.78	-
3	3.3	3	5.3	2	10,600	3280	0.69	66,900
4	3.3	2	5.3	1	1020	140	0.86	-
5	3.3	2	5.3	1	1020	150	0.85	-
6	3.3	3	5.3	1	1020	170	0.83	-
7	3.3	3	5.2	2	1020	210	0.79	20,700

*v_e_* and *v*_s_ are the flow rates of the aqueous phases in the extraction and stripping chambers, *d_e_* and *d_s_* are the distributor hole diameters in the extraction and stripping chambers, *x_f_* and *x_r_* are the concentration in the feed and raffinate, and *x_s_* is the concentration of bound phenol in the stripping phase at the end of the experiment, *E* = 1 − *x_r_*/*x_f_*.

**Table 3:** Conditions and results of experiments conducted in the large apparatus.

No	*v*_*e*_, L/h	*d_e_*, mm	*v*_s_, L/h	*d_s_*, mm	*x_f_*, ppm	*x_r_*, ppm	*E*	*x_s_*, ppm
1	3.3	2	5.3	1	1030	14.5	0.99	-
2	5.3	2	8.5	1	1030	18	0.98	10,350
3	7.0	2	11	1	1030	19	0.98	-
4	9.0	2	14	1	1030	35	0.97	17,800
5	3.3	3	5.3	2	1060	18	0.98	-
6	4.2	3	7.0	2	1060	13	0.99	-
7	5.3	3	8.5	2	1060	27	0.97	20,000
8	3.3	1	5.3	2	1090	20	0.98	-
9	5.3	1	8.5	2	1090	80	0.93	-
10	7.0	1	11	2	1090	180	0.84	17,700
11	3.3	1	5.3	2	10,600	380	0.96	-
12	5.3	1	8.5	2	10,600	4500	0.58	73,300
13	3.3	2	5.3	1	10,800	470	0.96	-
14	3.3	3	5.3	2	10,800	570	0.95	53,800
15	3.3	3	5.3	2	1060	11	0.99	-
16	4.2	3	11	2	1060	8	0.99	-

## Data Availability

Not applicable.
